# Time to sputum culture conversion and its predictors among patients with multidrug-resistant tuberculosis in Hangzhou, China: A retrospective cohort study

**DOI:** 10.1097/MD.0000000000023649

**Published:** 2020-12-11

**Authors:** Qingchun Li, Min Lu, Evelyn Hsieh, Limin Wu, Yifei Wu, Meng Wang, Le Wang, Gang Zhao, Li Xie, Han-Zhu Qian

**Affiliations:** a Hangzhou Center for Disease Control and Prevention, Hangzhou, Zhejiang Province, China; b Department of Internal Medicine, Yale School of Medicine, New Haven, CT; c SJTU-Yale Joint Center for Biostatistics and Data Science, Shanghai Jiao Tong University (SJTU), Shanghai, China; d Department of Biostatistics, Yale School of Public Health, New Haven, CT.

**Keywords:** China, multidrug-resistant tuberculosis, predictors, retrospective cohort, sputum culture conversion, treatment outcomes

## Abstract

The objective is to investigate the time to initial sputum culture conversion (SCC) and its predictors among multidrug-resistant tuberculosis (MDR-TB) patients in Hangzhou, China.

A retrospective cohort study was conducted among patients who initiated MDR-TB treatment from 2011 to 2015 in Hangzhou, China. Successful achievement of initial SCC was defined as 2 consecutive negative cultures taken at least 30 days apart after initiation of treatment of MDR-TB. Successful treatment outcomes included being cured and completing treatment, while poor treatment outcomes included treatment failure, loss to follow-up, and death. Time to initial SCC was analyzed using the Kaplan–Meier method, and Cox proportional hazards regression was used to identify predictors of SCC.

Among 384 patients enrolled with MDR-TB, 359 (93.5%) successfully achieved initial SCC after a median of 85 days (interquartile range, 40–112 days). A higher rate of SCC was observed in participants with successful treatment outcomes than those with poor treatment outcomes (*P*<.01). Multivariate analysis showed that age 25 to 64 years (compared with age<25; adjusted odds ratio [AOR], 0.7; 95% confidence interval [CI], 0.5–0.9; *P* *<* .01), age ≥65 years (compared with age < 25; AOR, 0.5; 95% CI, 0.3–0.8; *P* < .01), and household registration in Hangzhou (compared with non-Hangzhou registration; AOR, 1.3; 95% CI, 1.0–1.5; *P* *<* .05) were found to be associated with SCC.

Although high SCC and treatment success rates were observed among MDR-TB patients in Hangzhou, the prolonged duration to initial SCC underscores the importance of emphasizing measures for infection control. A new policy of shifting outpatient treatment to inpatient treatment in China may reduce the risk of transmission from patients in the time window prior to SCC.

## 1 Introduction

The World Health Organization (WHO) estimated that tuberculosis (TB) caused 1.6 million deaths in 2017, exceeding HIV globally as the infectious disease leading to the greatest number of deaths.^[1]^ In that same year, 10 million people developed TB disease with 9% of those cases occurring in China. Furthermore, 460,000 people worldwide were diagnosed with multidrug-resistant tuberculosis (MDR-TB), which is defined as resistance to at least 2 most common anti-TB drugs isoniazid and rifampin, and 13% of cases were in China.^[1]^ TB has been a major public health challenge in China, calling for the development of both broad coverage and high-quality programs for TB screening, diagnosis, treatment, and care, especially for individuals with MDR-TB.

Treatment of MDR-TB typically involves prolonged^[2]^ and expensive therapeutic regimens,^[1]^ with patients enduring at least 6 months of injections as well as side effects. The average cure rate of MDR-TB is only about 50% globally.^[1]^ Studies have shown that primary resistance, resulting from transmission of resistant TB strains, is more common than newly acquired drug resistance,^[3,4]^ and successful treatment can rapidly reduce the risk of transmission of MDR-TB to others.^[5]^ Therefore, effectively identifying and treating existing cases through high-quality diagnosis and treatment services is a crucial step in preventing the spread of MDR-TB.

Sputum culture conversion (SCC) is a prognostic marker of therapeutic efficacy in patients with MDR-TB.^[6–8]^ Additionally, better clinical outcomes have been observed among MDR-TB patients who achieve culture conversion.^[9,10]^ Based upon these findings, the WHO highly recommends routine SCC monitoring to assess treatment response and using 6-month SCC status as a proxy marker of final treatment outcome.^[2]^ Elucidating potential predictors of SCC in MDR-TB could help identify patients who are likely to develop poor outcomes and prompt clinical intervention to improve treatment outcomes for these individuals. Numerous studies have been conducted to investigate the predictors for MDR-TB treatment outcomes,^[11–13]^ but few have been carried out in China. We therefore designed a retrospective cohort study to evaluate the SCC and identify its predictors among MDR-TB patients in Hangzhou in eastern China.

## 2 Methods

### 2.1 Study overview

Hangzhou is the capital city of Zhejiang Province in eastern China. It comprises 13 districts, 1 county-level city and 2 counties, and has 7.2 million local residents and over 2 million migrant inhabitants. There are 13 TB specialty hospitals, with 2 designated for treating drug-resistant TB patients.

A retrospective cohort was designed among patients who were diagnosed with MDR-TB and initiated on treatment in one of the TB specialty hospitals in Hangzhou from 2011 to 2015. The eligibility criteria included: patients diagnosed with MDR-TB in one of the TB specialty hospitals in Hangzhou; initiated treatment after diagnosis; had demographic and clinical data, for example, gender, age, occupation, residence, ethnicity, year of starting TB treatment, sputum microscopy testing, culture and drug sensitivity testing, duration of therapy, and treatment outcome. All patients meeting these inclusion criteria were consecutively included in the cohort. The bacteriology examinations including sputum sample collection, smear, culture, and drug sensitivity tests have been reported elsewhere.^[14]^ TB culture was performed as follows: First, decontaminating and digesting the sputum with equal volume of 4% sodium hydroxide for 15 minutes; Second, inoculating 0.1 mL specimen into the Lowenstein-Jensen medium, and culturing it in an incubator at 37°C; then, observing the colony growth, which was confirmed by microscopic examination for AFB through Ziehl-Neelsen staining. Drug sensitivity test was performed using the proportion method on Löwenstein-Jensen medium, with the following concentrations: 0.2 micrograms per milliliter (μg/mL) for isoniazid, 2.0 μg/mL for ethambutol, 2.0 μg/mL for ofloxacin, 4.0 μg/mL for streptomycin, 30 μg/mL for kanamycin, and 40 μg/mL for rifampin. The critical growth proportion for drug resistance was 1% for all drugs. All drugs were obtained from Sigma Life Science Company (USA). The standard sensitive strain H37Rv was tested in each set of the tests and again within each set if the batch of medium was changed. The drug sensitivity test result or the H37Rv should be sensitive. All drug sensitivity tests were performed by the same staff in the TB reference laboratory at Hangzhou CDC.

Deidentified demographic, clinical, and sputum culture data, which were examined at a monthly interval, were extracted from the online Tuberculosis Information Management System (TBIMS).^[15]^ Because TB is a notifiable disease in China, all diagnosed cases are reported to TBIMS, and this database serves as a central repository for TB diagnosis, treatment, and monitoring data. All diagnosed MDR-TB patients who started treatment from 2011 to 2015 in Hangzhou, China were included in this analysis.

The study was approved by the ethics committee of Hangzhou City Center for Disease Control and Prevention.

### 2.2 Sputum culture conversion and treatment outcome

The outcome event was SCC, which was defined as 2 consecutive negative cultures taken at least 30 days apart following an initial positive culture. According to the WHO guidelines,^[16]^ treatment outcomes were defined as follows: A patient was considered cured if he/she completed treatment and had at least 5 consecutively negative sputum cultures during the final 12 months of treatment; or if one of those cultures was positive, then at least 3 subsequent consecutive cultures should have been negative. Treatment completion was defined as completion of MDR-TB treatments without evidence of failure, but with inadequate bacteriological records for classification as cure. Treatment failure was defined when 2 or more of the 5 final sputum cultures were positive, or 1 positive culture of the final 3 cultures during the final 12 months of treatment. Death was defined when a patient died due to any cause during treatment. A patient was considered lost to follow-up if treatment was interrupted for 2 or more consecutive months against his or her clinicians’ advice. These treatment outcomes were subsequently categorized into 2 groups: The *treatment success group* included patients who met criteria for cure or treatment completion, whereas the *poor treatment outcome group* included patients who were classified into the treatment failure, lost to follow-up or death categories.

### 2.3 Statistical analysis

Statistical analyses were conducted with SPSS software (IBM SPSS Statistics for Windows, version 19 [IBM Corp, Armonk, NY]). Proportions were computed for categorical variables and expressed as percentages. Means with standard deviations and medians with interquartile ranges (IQRs) were calculated for normally distributed and nonparametric continuous data, respectively. The Pearson *χ*^2^test or Fisher exact test was used to compare the differences in treatment outcomes between groups. Time to SCC was analyzed using the Kaplan–Meier method, and differences in survival times were assessed with the log-rank test. Bivariate and multivariable Cox proportional hazards regression analyses were used to identify predictors of SCC. Time of entry into cohort was the date of initiating treatment within the study period, and exit time was date of collecting the first negative sputum specimen for diagnosing SCC. Study participants were censored from the analysis at the time of death, last follow-up date, or December 31, 2015, depending on which came first.

All variables with *P* < .1 in univariate analysis were included in multivariable analysis. Statistical significance was defined as a 2-sided *P* value < .05.

## 3 Results

### 3.1 Demographic and clinical characteristics of the participants

Between 2011 and 2015, a total of 25,081 cases of pulmonary tuberculosis were diagnosed in Hangzhou, and 8806 were sputum smear positive and screened for drug resistance. Among 527 patients diagnosed with MDR-TB, 394 (74.8%) started treatment during the study period. Patients who did not start treatment during the study period were excluded from this study. Of the 394 patients, 5 patients who were still on treatment at the end of study, and 5 patients who had incomplete information on treatment and treatment outcome were excluded from the study, resulting in a total of 384 patients with MDR-TB included in the analysis.

Of the 384 participants, 296 (77.1%) met criteria for successful treatment outcomes and 88 (22.9%) had poor treatment outcomes. The mean age of participants was 41.7 year (standard error, 15.4). The median duration of follow-up was 86.5 days (range, 13–1281; IQR, 44.5–117.5). Table 1 displays demographic and clinical characteristics of the participants.

**Table 1:** Demographic and clinical characteristics of study participants.

		Treatment outcomes			
Variables	N (%)	Successful, N (%)	Poor, N (%)	χ2	*P* value
Total	384	296 (77.1)	88 (22.9)		
Gender				5.36	<.05
Male	272 (70.8)	201 (67.9)	71 (80.7)		
Female	112 (29.2)	95 (32.1)	17 (19.3)		
Age, y				14.4	<.05
<25	58 (15.1)	55 (18.6)	3 (3.4)		
25≤age<65	293 (76.3)	220 (74.3)	73 (83.0)		
Age>65	33 (8.6)	21 (7.1)	12 (13.6)		
Ethnic				0.14	.99
Han	378 (98.4)	291 (98.3)	87 (98.9)		
Other	6 (1.6)	5 (1.7)	1 (1.1)		
Occupation				7.83	<.05
Other	101 (26.3)	88 (29.7)	13 (14.8)		
Farmer/worker/migrant worker	283 (73.7)	208 (70.3)	75 (85.2)		
Residence				6.48	<.05
Rural	160 (41.7)	113 (38.2)	47 (53.4)		
Urban	224 (58.3)	183 (61.8)	41 (46.6)		
Household registration in Hangzhou				0.52	.47
No	214 (55.7)	162 (54.7)	52 (59.1)		
Yes	170 (44.3)	134 (45.3)	36 (40.9)		
Resistance to ethambutol				0.29	.59
No	219 (57)	171 (57.8)	48 (54.6)		
Yes	165 (43)	125 (42.2)	40 (45.5)		
Resistance to streptomycin				0.11	.74
No	147 (38.3)	112 (37.8)	35 (39.8)		
Yes	237 (61.7)	184 (62.2)	53 (60.2)		
Resistance to ofloxacin				0.16	.69
No	354 (92.2)	272 (91.9)	82 (93.2)		
Yes	30 (7.8)	24 (8.1)	6 (6.8)		
Resistance to kanamycin				0.01	.99
No	375 (97.7)	289 (97.6)	86 (97.7)		
Yes	9 (2.3)	7 (2.4)	2 (2.3)		
Number of resistant drugs				0.18	.67
2	98 (25.5)	74 (25.0)	24 (27.3)		
>2	286 (74.5)	222 (75.0)	64 (72.7)		
Previous TB treatment history				2.81	.09
No	29 (7.6)	26 (8.8)	3 (3.4)		
Yes	355 (92.4)	270 (91.2)	85 (96.6)		

### 3.2 Sputum culture conversion and treatment outcomes

**Figure 1:**
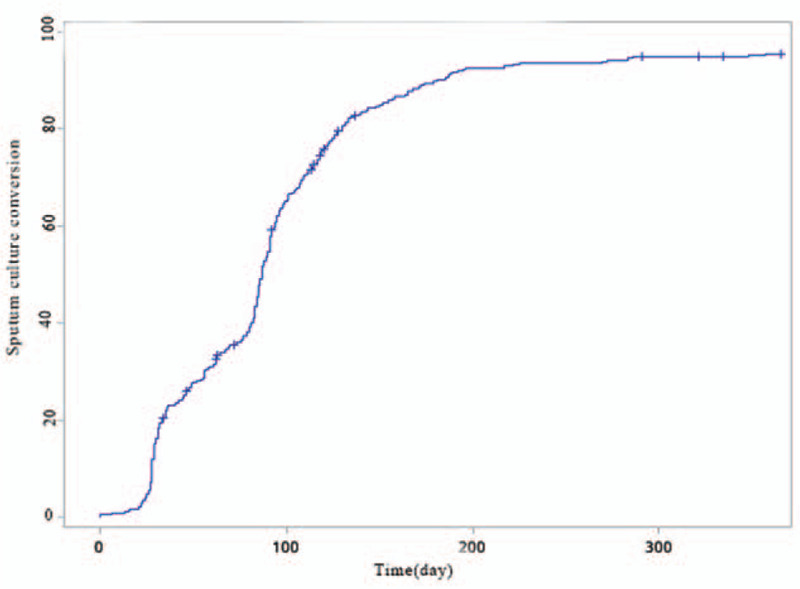
Survival curve during the first 12 months of treatment.

In all, 359 (93.5%) patients achieved SCC in a median of 85 days (range, 13–541; IQR, 40–112), and 25 (6.5%) failed to achieve SCC. Figure 1 shows the survival curve during the first 12 months of treatment, and there is little change thereafter.

**Figure 2:**
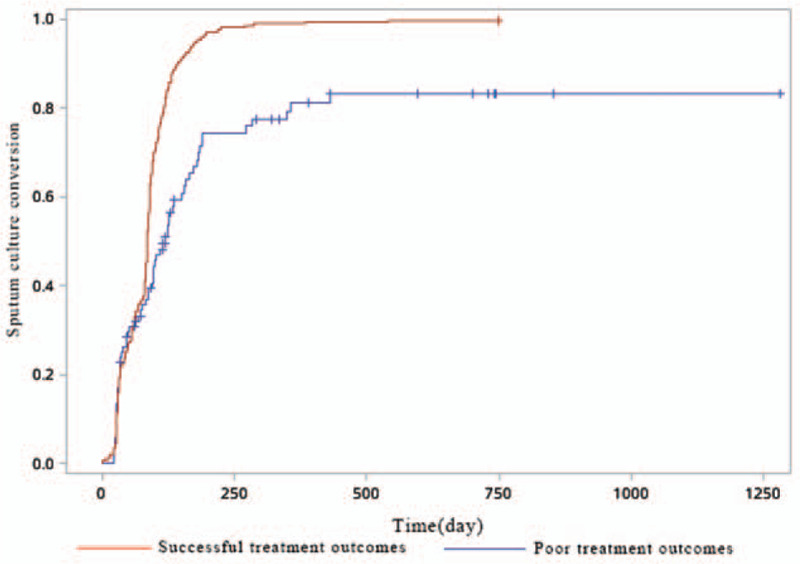
Time to initial SCC between groups with different treatment outcomes. SCC = sputum culture conversion.

The observed rate of SCC was markedly higher among participants with successful treatment outcomes compared with those with poor treatment outcomes (odds ratio [OR], 110.6; 95% confidence interval [CI], 14.7–832.7; *P* < .01) (Table 2). The time to initial SCC also differed significantly between those with successful treatment outcomes and those with poor treatment outcomes (χ^2^=35.5, *P* < .01; Fig. 2).

**Table 2:** Concordance of SCC and treatment outcomes.

	Treatment outcome			
SCC	Successful, N (%)	Poor, N (%)	χ^2^	*P* value
Yes	295 (99.7)	64 (72.7)	80.9	<.001
No	1 (0.3)	24 (27.3)		

### 3.3 Predictors of time to initial sputum culture conversion

All variables with *P* < .1 in univariate analysis (Table 3), including age ≥65 years (OR, 0.5; 95% CI, 0.3–0.8; *P* *<* .01), age 25–64 years (OR, 0.7; 95% CI, 0.5–0.9; *P* < .01), occupation (OR, 0.8; 95% CI, 0.6–1.0; *P* < .05), and household registration in Hangzhou (OR, 1.2; 95% CI, 1.0–1.5; *P*=.09), were entered in multivariable analysis. Variables that remained in the final model included: age 25 to 64 years (AOR, 0.7; 95% CI, 0.5–0.9; *P* < .01), age ≥65 years (AOR, 0.5; 95% CI, 0.3–0.8; *P* < .01), and household registration in Hangzhou (AOR, 1.3; 95% CI, 1.0–1.5; *P* < .05) (Table 4).

**Table 3:** Univariate analysis of predictors of SCC among MDR-TB patients in Hangzhou, China.

Variable	N	Sputum culture conversion (%)	HR (95% CI)	*P* value
Residence				
Rural	160	143 (89.4)		
Urban	224	216 (96.4)	1.0 (0.8,1.2)	.97
Household registration in Hangzhou				
No	214	199 (93.0)		
Yes	170	160 (94.1)	1.2 (1.0,1.5)	.09
Gender				
Male	272	252 (92.6)		
Female	112	107 (95.5)	1.0 (0.8,1.3)	.71
Age, y				
<25	58	58 (100.0)		
25–64	293	273 (93.2)	0.7 (0.5,0.9)	<.01
≥65	33	28 (84.8)	0.5 (0.3,0.8)	<.01
Ethnicity				
Han	378	354 (93.7)		
Other	6	5 (83.3)	1.1 (0.5,2.7)	.83
Occupation				
Other	101	100 (99.0)		
Farmer/worker/migrant worker	283	259 (91.5)	0.8 (0.6,1.0)	.03
Previous TB treatment				
No	29	27 (93.1)		
Yes	355	332 (93.5)	1.0 (0.7,1.5)	.85
Number of resistant drugs				
2	98	95 (96.9)		
>2	286	264 (92.3)	0.9 (0.7,1.2)	.44
Resistance to ethambutol				
No	219	209 (95.4)		
Yes	165	150 (90.9)	0.9 (0.7,1.1)	.29
Resistance to streptomycin				
No	147	139 (94.6)		
Yes	237	220 (92.8)	0.9 (0.8,1.2)	.51
Resistance to ofloxacin				
No	354	332 (93.8)		
Yes	30	27 (90.0)	0.8 (0.5,1.2)	.32
Resistance to kanamycin				
No	375	350 (93.3)		
Yes	9	9 (100.0)	1.1 (0.6,2.1)	.85

CI = confidence interval, HR = hazard ratio.

**Table 4:** Cox regression analysis of predictors of SCC among MDR-TB patients in Hangzhou, China.

Variable	AHR (95% CI)	*P* value
Household registration in Hangzhou		
No	1.0	
Yes	1.3 (1.0,1.5)	<.05
Age, y		
<25	1.0	
25–64	0.7 (0.5,0.9)	<.01
≥65	0.5 (0.3,0.8)	<.01

AHR = adjusted hazard ratio, CI = confidence interval.

## 4 Discussion

Routine sputum culture monitoring during clinical treatment is a critical component of successful MDR-TB management,^[2,12]^ as SCC has been demonstrated consistently to predict the therapeutic efficacy of treatment, and time to SCC has been shown to be early predictor of treatment outcomes.^[8,17]^ Few studies have evaluated these issues in China, and this study evaluated time to SCC and predictors of initial SCC among patients with MDR-TB in Hangzhou and has several important findings.

First, the present study revealed that 93.5% participants achieved SCC in their treatment course. To the best of our knowledge, this is the highest SCC rate reported in the past 5 years. The SCC rates reported by studies from Pakistan,^[7]^ Ethiopia,^[11,12]^ Peru,^[18]^ India,^[19]^ Jiangsu province in China,^[8]^ and 1 multicenter study carried out in 9 countries^[6]^ varied from 76.3% to 90.0%. Our study showed a higher SCC rate than reported in Chinese^[20–22]^ and most international studies,^[6,23–25]^ and the high SCC rate is also concordant with a high treatment success rate. Our previous study in the same population also found a decline trend of drug resistance prevalence over the years 2011 to 2015: resistance to any first-line drugs from 31.3% to 22.3% and MDR from 11.6% to 8.0%. These findings suggested a significant progress achieved by TB treatment and management programs in Hangzhou.^[14]^

Second, a higher SCC rate was observed among participants with successful treatment outcomes compared with those with poor treatment outcomes, which is similar to results reported in previous studies using SCC as a prognostic marker for treatment outcomes in patients with MDR-TB.^[6–8]^ However, further research should investigate the validity of SCC at different timepoints as an early predictor of treatment efficacy, as it may differ by regions with different HIV prevalence, capacities for MDR-TB treatment, and drug resistance patterns.^[12,13]^ For example, the 6-month SCC had stronger association with cure than 4-month SCC among Pakistan patients,^[7]^ while studies conducted in China and Ethiopia showed that the optimum time point of achieving SCC for predicting successful treatment outcome was between 4 and 6 months.^[26]^

Third, the median time to initial SCC in our study was 85 days, which is in the upper range of 58 to 91.5 days as reported in earlier studies.^[7,8,11,12,18,19]^ Factors influencing the duration of time to SCC may include treatment regimens, second-line drug susceptibility, loss to follow-up rate, and sensitivity of laboratory testing. Further research is needed on identifying these factors.

MDR-TB patients with positive sputum cultures are infectious and could potentially act as a source of transmission of disease in the community.^[12]^ This has been reported as the main driver of MDR-TB in China, as opposed to de novo acquisition of drug resistance.^[4]^ The rates of SCC and treatment success were high, but the median time from starting treatment to initial SCC was near 3 months, and these mixed results indicated that further efforts should be made to reduce the time to SCC for the purpose of effective infection control. Most Chinese MDR-TB patients receive outpatient treatment without isolation from their families and communities, except for those with severe side effects or comorbidities who receive inpatient treatment, so few measures have been taken for infection control in routine treatment and management of MDR-TB patients, this consequently increases the risk of transmission. Hangzhou health department is planning to provide free inpatient treatment for MDR-TB patients until they achieve SCC. This new policy could help reduce the likelihood of MDR-TB transmission to their contacts.

Older patients were less likely to achieve SCC, and this finding is in line with previous studies reporting older age as a risk factor for poor treatment outcomes among patients with MDR-TB.^[27,28]^ Furthermore, patients with household registration in Hangzhou were more likely to achieve SCC. One reason for this may be that migrant patients without household registration were more likely to have treatment interruption due to limited insurance coverage and unstable living place, and financial resources have been reported to be strongly associated with treatment outcomes.^[29]^ Measures should be taken to prevent the transmission of MDR-TB among these subgroups of older patients and non-Hangzhou registered residents before they became sputum culture conversion.

This study has several limitations. First, certain important clinical variables such as body mass index, HIV infection status, smoking, and alcohol use, which have been reported to be related to initial SCC,^[12,22]^ were not investigated in this study. These data were not collected or uploaded to TBIMS by hospitals. Second, patients were asked to undergo sputum examinations once a month according to the guidelines for treatment of drug-resistant tuberculosis in China,^[30]^ but most patients have missed 1 or more examination, which may affect the accuracy of the observed time to initial SCC in this study. Third, this study was carried out in a single; therefore, the findings are not necessarily generalizable to other cities.

However, this retrospective study is among few studies evaluating the rate and predictors of initial SCC among MDR-TB patients in China and provides valuable data on high rate of initial SCC and its correlation with successful treatment outcome. This study also highlights the need for revising the current policy of outpatient treatment to reduce the risk of transmission during the window from initiating treatment to achieving SCC.

## Author contributions

**Conceptualization:** Qingchun Li, Han-Zhu Qian.

**Formal analysis:** Qingchun Li, Evelyn Hsieh.

**Funding acquisition:** Qingchun Li.

**Investigation:** Qingchun Li, Min Lu, Limin Wu, Yifei Wu, Meng Wang, Le Wang.

**Laboratory tests:** Yifei Wu

**Project administration:** Li Xie.

**Supervision:** Gang Zhao, Han-Zhu Qian.

**Writing – original draft:** Qingchun Li.

**Writing – review & editing:** Evelyn Hsieh, Han-Zhu Qian.
